# Integrating Multi-Source Data for Aviation Noise Prediction: A Hybrid CNN–BiLSTM–Attention Model Approach

**DOI:** 10.3390/s25165085

**Published:** 2025-08-15

**Authors:** Yinxiang Fu, Shiman Sun, Jie Liu, Wenjian Xu, Meiqi Shao, Xinyu Fan, Jihong Lv, Xinpu Feng, Ke Tang

**Affiliations:** 1 Chongqing Airport Group Co., Ltd., Chongqing 401120, China; 18983287870@163.com (Y.F.); 15310850011@163.com (J.L.); fanxinyu199911@outlook.com (X.F.); 2 Civil Aviation Research Base (Beijing) Co., Ltd., Beijing 100621, China; jxcrssm@163.com (S.S.); fengxinpu@163.com (X.F.); 3 China Airport Construction Group Co., Ltd., Beijing 100621, China; 4 School of Air Traffic Management, Civil Aviation Flight University of China, Guanghan 618307, China; xuwenjian0114@163.com (W.X.); shaomq2024@163.com (M.S.)

**Keywords:** CNN–BiLSTM–Attention, multi-source data integration, spatiotemporal alignment, aviation noise prediction, IDW

## Abstract

Driven by the increasing global population and rapid urbanization, aircraft noise pollution has emerged as a significant environmental challenge, impeding the sustainable development of the aviation industry. Traditional noise prediction methods are limited by incomplete datasets, insufficient spatiotemporal consistency, and poor adaptability to complex meteorological conditions, making it difficult to achieve precise noise management. To address these limitations, this study proposes a novel noise prediction framework based on a hybrid Convolutional Neural Network–Bidirectional Long Short-Term Memory–Attention (CNN–BiLSTM–Attention) model. By integrating multi-source data, including meteorological parameters (e.g., temperature, humidity, wind speed) and aircraft trajectory data (e.g., altitude, longitude, latitude), the framework achieves high-precision prediction of aircraft noise. The Haversine formula and inverse distance weighting (IDW) interpolation are employed to effectively supplement missing data, while spatiotemporal alignment techniques ensure data consistency. The CNN–BiLSTM–Attention model leverages the spatial feature extraction capabilities of CNNs, the bidirectional temporal sequence processing capabilities of BiLSTMs, and the context-enhancing properties of the attention mechanism to capture the spatiotemporal characteristics of noise. The experimental results indicate that the model’s predicted mean value of 68.66 closely approximates the actual value of 68.16, with a minimal difference of 0.5 and a mean absolute error of 0.89%. Notably, the error remained below 2% in 91.4% of the prediction rounds. Furthermore, ablation studies revealed that the complete CNN–BiLSTM–AM model significantly outperformed single-structure models. The incorporation of the attention mechanism was found to markedly enhance both the accuracy and generalization capability of the model. These findings highlight the model’s robust performance and reliability in predicting aviation noise. This study provides a scientific basis for effective aviation noise management and offers an innovative solution for addressing noise prediction problems under data-scarce conditions.

## 1. Introduction

Long-term nocturnal exposure to specific levels of noise can induce a spectrum of adverse health effects [1,2]. Biologically, noise exposure exerts substantial negative impacts on human health, prominently manifesting as sleep disturbances, psychological stress, cardiovascular diseases, and metabolic system dysfunction [3]. Notably, both traffic noise and wind turbine noise are significantly correlated with these health issues [4]. Thus, noise pollution, an often-neglected environmental concern, warrants serious attention due to its potential severity. To alleviate the detrimental effects of noise on human health, it is crucial to adopt technological innovations and implement comprehensive management strategies [5].

In traffic noise prediction research, statistical regression models and deep learning techniques based on variables like traffic volume, vehicle speed, and road type are widely used. For example, a comprehensive framework integrating land use regression (LUR) modeling with real-time traffic data has been developed to predict environmental noise levels in specific areas. This addresses the challenges of assessing community environmental noise exposure due to the lack of conventional noise monitoring data or validated prediction methods [6]. Monte Carlo simulation techniques have been employed to generate noise prediction models for specific vehicle locations based on random probability distributions of vehicles, with traffic noise calculated according to the principle of energy superposition [7]. Traffic noise prediction models based on shoulder equivalent sound sources have improved the noise source strength model using sound pressure level data and attenuation laws, offering a more efficient method for traffic noise assessment [8]. Noise prediction models customized using the CORTN method for different types of intersections have outperformed general models, providing more accurate fits to traffic volume and site characteristic data [9]. An integrated model combining mechanism-driven models with data-driven techniques has been developed to predict vehicle road noise. This aims to overcome the limitations of mechanism analysis and the strict data quality requirements of data-driven techniques, thereby improving the accuracy of in-vehicle noise prediction [10]. Innovative stochastic deep learning (SDL) noise prediction models have been proposed for construction site noise prediction, providing an efficient tool for predicting noise levels from stochastic data [11]. A novel deep learning model based on autoencoders and long short-term memory (LSTM) networks, with optimized hyperparameter combinations using grid search methods, has been developed for predicting environmental noise levels [12]. These noise prediction studies primarily focus on the characteristics of noise sources in different physical environments. However, existing research on noise source modeling and prediction is typically conducted under static or fixed conditions, neglecting dynamic environmental factors such as meteorological elements.

Aircraft noise, a pervasive source of noise pollution in daily life, significantly contributes to environmental degradation and has emerged as a primary constraint on the expansion of major airports worldwide. This, in turn, impacts the sustainable development of the aviation market. To tackle the issue of aircraft noise effectively, various noise prediction technologies have been developed. Physical models offer prediction methods grounded in fundamental physical principles. Machine learning techniques, through extensive data training, enable automated and precise predictions. Multi-source data fusion techniques integrate information from diverse sources to further improve the accuracy and comprehensiveness of noise predictions.

Physical models are developed based on actual noise sources and their propagation mechanisms, simulating noise generation and diffusion through mathematical equations. These models are generally well-suited for noise prediction under known physical conditions. For example, the prediction model for aircraft slat noise is typically grounded in the first principles of aerodynamic sound generation theory and source flow physics [13]. In cases where the geometric shape of an object varies, finite element modeling can be utilized for comprehensive parametric studies to assess how deviations from a standard cross-section impact ground noise and vibration predictions [14]. The wave-based acoustic network model, which reformulates linear acoustics into state-space form, can be employed to investigate the stability of thermoacoustic systems in both the frequency and time domains. More accurate methods have also been developed for the extended analysis of time domain techniques [15]. The finite-difference wave number–time domain method for acoustic field prediction in a uniformly moving medium has demonstrated strong performance for accurate and flexible time-varying acoustic field predictions [16]. The wave number domain acoustic finite element (2.5D acoustic FE) method can effectively reduce the research workload associated with acoustic problems [17,18].

Machine learning techniques do not rely on strict physical laws but instead learn the patterns of noise automatically through training on large amounts of historical data, thereby making predictions [19]. This approach is particularly effective when dealing with large volumes of data or complex variations. A prediction model based on multivariate regression analysis and a two-layer fuzzy neural network (MRA-2LFNN) can predict the fan noise of a train’s electric traction system, achieving an average prediction accuracy of 94.15% [20]. The random forest (RF) algorithm can be employed to predict the power spectral density (PSD) and overall sound pressure level (OASPL) distribution on the supercritical airfoil RAE2822. Although the RF algorithm may not provide high-precision prediction probabilities at all monitoring points, it is capable of addressing the prediction of airfoil aerodynamic noise [21]. Additionally, both random forest (RF) and long short-term memory (LSTM) machine learning methods can predict aircraft noise at lateral, flyover, and approach points based on maximum takeoff mass (MTOM), maximum landing mass (MLM), and engine takeoff thrust. Comparing the results, LSTM achieves more accurate noise modeling [22]. Moreover, a wind turbine noise prediction model based on random forest regression (Random Forest Regression) can be constructed using systematic data and on-site sound pressure level measurements, with the random forest model demonstrating high accuracy in general noise level predictions [23]. In the field of noise prediction, the combination of aerodynamic principles and deep neural networks is common. The physics-guided neural network (PGNN) addresses the limitations of traditional physical models in terms of prediction accuracy and the generalization capability of data-driven models, creating a model that retains the stability of physical models while enhancing the accuracy of data-driven models [24]. When only radar or ADS-B flight surveillance data are available, neural network training can accurately infer an aircraft’s takeoff weight, thrust profile, and flap settings [25]; these key flight parameters can also serve as inputs to drive neural network–based noise prediction models, thereby enhancing the accuracy and practicality of aircraft noise assessment.

Research on airfoil and aerodynamic noise prediction models is of significant importance for the optimization of aircraft design and the reduction in flight noise pollution. Many researchers have employed a variety of prediction methods, such as aerodynamic noise prediction based on physical models and empirical formulas, and they have achieved certain progress in the field of aircraft noise control. For the prediction of trailing-edge noise from aircraft airfoils, methods can be categorized into semi-empirical, direct, and hybrid approaches, each with its specific advantages and applicable scenarios [26]. Engineering noise prediction tools based on mathematical models can accurately assess the noise reduction effects of serrated airfoils and reveal the advantages of new models in considering boundary layer characteristics and acoustic wave interference effects by comparing with CFD simulations and experimental data [27]. Combining airfoil noise models with CFD calculations, and considering inflow turbulence and airfoil self-noise mechanisms, can improve the accuracy of predicting aerodynamic broadband noise generated by vertical-axis wind turbines [28]. Using the random forest model for rapid assessment of aerodynamic noise levels can address the issues of long computational cycles and high experimental measurement costs, and frequency domain models perform better than time domain models in predicting aerodynamic noise [29]. Although existing prediction models provide an important basis for theoretical research, considering the influence of more external factors such as meteorological conditions and flight paths, this study aims to further improve the reliability and accuracy of noise prediction by integrating these factors.

In recent years, Internet of Things (IoT)-related Application Programming Interfaces (APIs) have experienced rapid development across various fields [30]. Particularly, IoT applications leveraging heterogeneous wireless sensor networks have garnered significant attention [31]. As IoT technology continues to mature, the potential of real-time monitoring technology in noise management and predictive analysis has become increasingly evident. The integration of predictive models with IoT technology enables effective assessment of air quality and noise levels in environmental settings [32].

Despite extensive research into the prediction modeling of aircraft noise, the majority of existing methods concentrate predominantly on singular factors, such as engine parameters, flight phases, or airport layout, while neglecting the compounded impacts of critical environmental factors, including meteorological conditions and flight path variations. Meteorological elements, namely wind speed, wind direction, air temperature, and atmospheric stability, are known to exert a substantial influence on the propagation pathways of noise. Concurrently, the spatial distribution of flight trajectories directly governs the location and intensity of noise sources. Regrettably, these factors are frequently oversimplified or not methodically integrated into the modeling frameworks presented in the extant literature, thereby constraining the prediction accuracy and adaptability of the models. Consequently, there is a pressing need to develop a multi-source data prediction model that amalgamates meteorological and trajectory features, thereby augmenting the practicality and accuracy of noise prediction in complex operational scenarios.

In this context, this study utilized real-time monitoring data from Chongqing Jiangbei International Airport to systematically analyze sensor data collected from multiple monitoring points around the airport. The dataset encompasses meteorological parameters (e.g., temperature, humidity, wind speed) and flight trajectory data (e.g., aircraft altitude, longitude, latitude). To construct the noise prediction model, a hybrid model combining Convolutional Neural Network (CNN) and Bidirectional Long Short-Term Memory (BiLSTM) network with an attention mechanism (CNN–BiLSTM–Attention) was employed. This model effectively captures complex temporal features and achieves high-precision noise prediction through the integration of meteorological and flight trajectory data. The primary aim of this study is to provide a scientific basis for airport noise management and to elucidate the impact mechanisms of meteorological and flight trajectory factors on airport noise levels. It is anticipated that this study will offer valuable decision-making support for the development of noise control strategies and provide theoretical references for future research in related fields.

## 2. Methods for Noise Assessment and Simulation

### 2.1. Metrics for Single-Event Evaluation Based on Loudness

Loudness, serving as a measure of sound intensity, amalgamates the physical attributes of sound with the physiological responses elicited in the listener. This dual integration offers a holistic portrayal of noise intensity, spanning both objective and subjective dimensions. The present paper delves into single-event evaluation metrics that are predicated on loudness, with a particular emphasis on three cardinal indicators: the maximum A-weighted sound level, the equivalent continuous A-weighted sound level, and the A-weighted sound exposure level.

The equivalent continuous A-weighted sound level ($L_{\mathrm{Aeq}}$) is defined as the average sound level over a specified measurement period, calculated by substituting the energy of non-steady-state noise with that of a steady-state noise possessing the same total energy. The precise formulation is presented in Equation (1).

(1) $$L_{\mathrm{Aeq}}=10\times \mathrm{lg}(\frac{1}{T}\int_{0}^{T}10^{0.1\times L_{A}}\mathrm{dt})$$

where T represents the duration of the noise, while $L_{A}$ denotes the A-weighted sound level measured at a specific moment within the time interval T.

The A-weighted sound exposure level (Sound Exposure Level, SEL), also referred to as the sound exposure level, represents the average A-weighted sound level over a 1 s period, equivalent to the total noise energy received at a specific measurement point during a given noise exposure duration. The A-weighted sound exposure level can be calculated using Equation (2).

(2) $$L_{\mathrm{SEL}}=10\times \mathrm{lg}(\frac{1}{T_{0}}\int_{T_{1}}^{T_{2}}10^{0.1\times L_{A}}\mathrm{dt})$$

where $T_{0}$ is the specified measurement time for the A-weighted sound level, which can be taken as 1 s, $T_{1}$ is the start time of a single noise event, $T_{2}$ is the end time of a single noise event, and $L_{A}$ is the A-weighted sound level measured at a particular moment within the noise duration.

The maximum A-weighted sound level (Maximum A-weighted Sound Level, $L_{\max}$) refers to the peak A-weighted sound level measured at a specific point during a given measurement period. While $L_{\mathrm{Aeq}}$ can represent the average intensity of noise and SEL can reflect the overall exposure of a single noise event, $L_{\max}$ highlights the instantaneous maximum value of the noise. Together, these metrics provide a comprehensive noise assessment from three aspects: duration, total event exposure, and instantaneous intensity.

### 2.2. Automatic Dependent Surveillance-Broadcast (ADS-B)

ADS-B is an advanced air traffic surveillance technology that acquires precise aircraft position information via satellite navigation systems, such as the Global Positioning System (GPS), and broadcasts this data in real-time to ground stations and other aircraft through radio signals. ADS-B operates in two modes: ADS-B Out, where aircraft transmit their flight status information, and ADS-B In, where aircraft receive information from other aircraft and ground stations. Compared to traditional radar surveillance, ADS-B provides superior positioning accuracy and broader coverage, particularly in remote areas where radar coverage is limited. Additionally, ADS-B enhances the efficiency and safety of air traffic management by facilitating information exchange between aircraft. In recent years, many countries and regions have required new aircraft to be equipped with ADS-B devices, especially in high-altitude flight zones. These broadcast signals include key flight parameters such as real-time position, altitude, speed, and heading, with high update rates and reliable accuracy, offering essential dynamic flight information for multi-source data integration [33].

### 2.3. Aviation Environmental Design Tool (AEDT)

**Figure 1:**
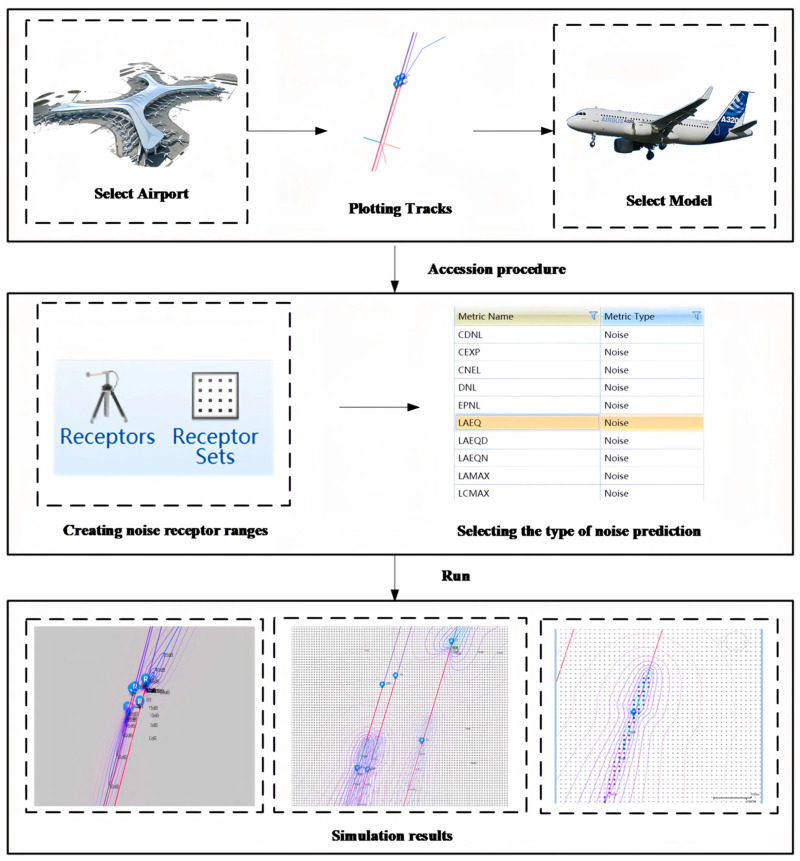
The specific operational procedures of the AEDT.

Noise contours serve as essential tools for predicting and mitigating noise annoyance. They provide critical information for land-use planning guidelines and regulations, as well as other strategies aimed at reducing noise annoyance [34]. The Aviation Environmental Design Tool 3d(AEDT V3d) is a specialized software system designed to simulate aircraft operating procedures and calculate the noise impacts generated during the takeoff and landing processes of single or multiple flights. The aircraft performance data utilized by AEDT primarily originates from the International Civil Aviation Organization (ICAO) Aircraft Noise and Performance (ANP) database and the Base of Aircraft Data (BADA). AEDT is capable of accurately assessing the propagation characteristics of noise during flight operations and is well-suited for average noise assessments in complex scenarios, such as annual aviation operations. The quality of the input data has a significant impact on the prediction accuracy. The specific operational procedures are illustrated in Figure 1.

**Figure 2:**
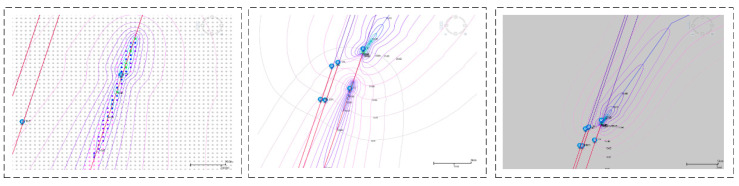
Map of noise distribution contours.

This study employs Chongqing Jiangbei International Airport as a case study. By referencing the airport’s aeronautical charts, the precise latitude and longitude coordinates of the relevant waypoints are initially established. The Airbus A320 aircraft is selected as the subject for simulating noise distribution during its five-edge approach and departure on RWY03, with an emphasis on the noise intensity and its variation patterns at different flight stages. To achieve accurate noise impact prediction, multiple grid monitoring points are strategically placed within the noise-affected area of the airport terminal region. Through simulation calculations, the aircraft noise levels at each monitoring point under the central flight track are obtained. Based on these simulation results, the noise distribution contour map is presented in Figure 2.

Figure 2 illustrates the $L_{\mathrm{Aeq}}$ noise distribution contour map for a single A320 aircraft during takeoff and landing operations. In this figure, noise levels below 40 dB are indicated by gray dots; levels between 40 and 45 dB are shown in pink; levels from 45 to 50 dB are depicted in purple; levels from 50 to 55 dB are marked by blue dots; levels between 55 and 60 dB are green; levels from 60 to 65 dB are yellow; levels ranging from 65 to 70 dB are orange; and levels exceeding 70 dB are highlighted in red. The aggregation of these color-coded dots delineates the extent of noise impact and forms the basis for the noise distribution contour lines.

By visualizing the spatial distribution of noise during takeoff and landing, we can intuitively reveal the spatial characteristics of noise distribution, particularly the variations in noise intensity at different locations and times. These visualizations provide a critical basis for studying the spatiotemporal characteristics of noise sources.

## 3. Data Selection and Processing

**Figure 3:**
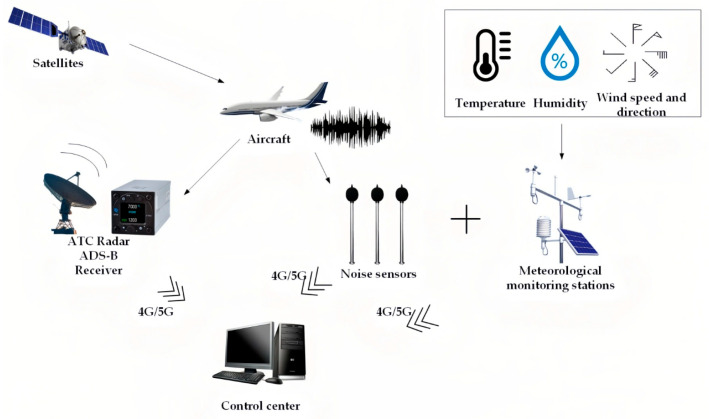
Diagram of the prediction model architecture.

This study develops a noise prediction model that integrates a wide range of meteorological factors, and the architecture of this model is depicted in Figure 3.

### 3.1. Placement of Monitoring Stations

**Figure 4:**
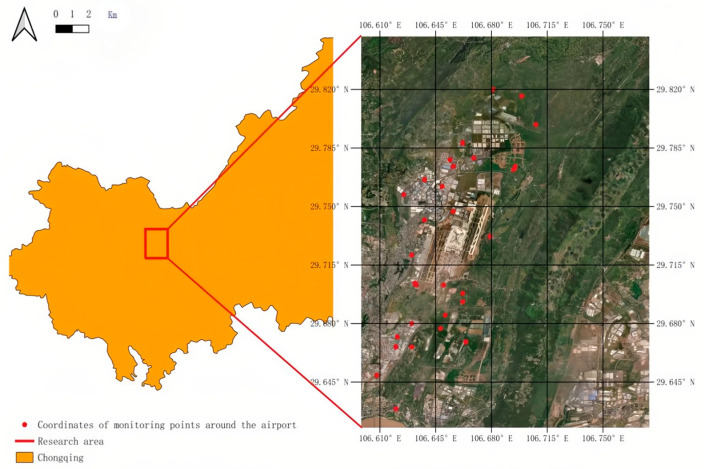
Map of airport noise monitoring station deployment.

This study collected data from 30 monitoring stations situated around Chongqing Jiangbei International Airport, with their geographic locations depicted in Figure 4. These stations are equipped with high-precision sensors that facilitate real-time collection of multidimensional environmental data, covering key meteorological parameters such as temperature, humidity, wind speed, and precipitation. Meteorological data are sourced from the meteorological stations associated with each monitoring site, ensuring the timeliness and accuracy of the data. The wide distribution of the monitoring stations enables comprehensive capture of the meteorological conditions in the airport’s surrounding area, providing essential environmental variables for subsequent noise prediction. Additionally, this study integrates aircraft flight data obtained via the ADS-B (Automatic Dependent Surveillance-Broadcast) system, including information on flight altitude, longitude, and latitude. These data, characterized by high temporal resolution and spatial accuracy, offer critical dynamic flight parameters for the noise prediction model. To address the heterogeneity of these data, this study employs multi-source data fusion techniques to process and integrate data from different sources, resolving discrepancies in format, precision, and spatiotemporal scales.

### 3.2. Data Preprocessing

#### 3.2.1. Data Imputation

This study utilized 24 h monitoring data from 3:00 to 15:00 on 9 January 2025, and from 9:00 to 21:00 on 14 January 2025, to form the initial dataset. However, this initial dataset exhibited some missing data points. To rectify this, during the preprocessing of meteorological data, the study prioritized ensuring the integrity of key meteorological variables, including temperature, wind speed, precipitation, and humidity. Given that the spatial locations of the monitoring points are defined by latitude and longitude coordinates, the study employed the Haversine formula in conjunction with inverse distance weighting (IDW) interpolation to effectively impute the missing data.

Considering that the original dataset is grounded in a geographic coordinate system, the Haversine formula is essential for calculating the spherical distance between two points, with the specific calculation detailed in Equation (3).

(3) $$d(x_{0},y_{0},x_{i},y_{i})=2R\cdot \arcsin(\sqrt{\sin^{2}(\frac{\Delta \emptyset }{2})+\cos(\emptyset_{0})\cdot \cos(\emptyset_{i})\cdot \sin^{2}(\frac{\Delta \lambda }{2})})$$

where R denotes the Earth’s radius, $\Delta \emptyset =\emptyset_{0}-\emptyset_{i}$ represents the latitude difference between the target and known points, $\Delta \lambda =\lambda_{0}-\lambda_{i}$ represents the longitude difference between the target and known points, $\emptyset_{0},\lambda_{0}$ represents the target point’s latitude and longitude, and $\emptyset_{i},\lambda_{i}$ represents the known point’s latitude and longitude.

**Figure 5:**
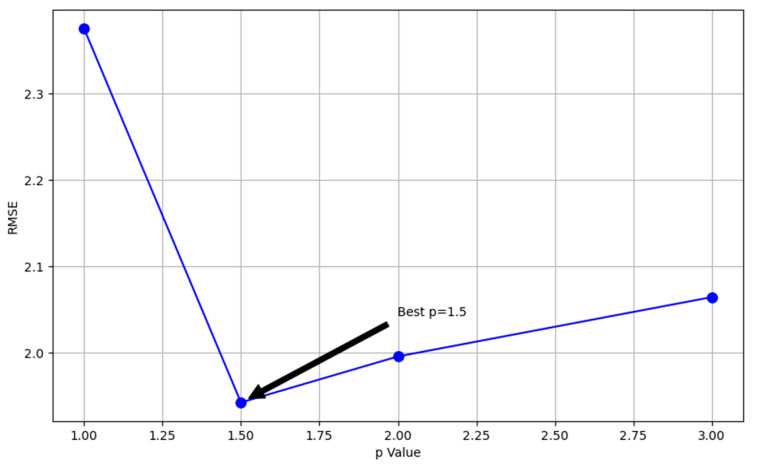
IDW interpolation RMSE comparison.

Next, the target points are selected. For each missing data point, its adjacent known data points are identified. Interpolation is then performed based on the principle that the closer a known point is to the target point, the greater its weight. This process is repeated until all missing data are filled, ensuring that the local characteristics and spatial consistency of the data are maintained, as shown in Equation (4).

(4) $$T(x_{0},y_{0})=\frac{\sum_{i=1}^{n}\frac{T(x_{i},y_{i})}{d{\left(x_{0},y_{0},x_{i},y_{i}\right)}^{p}}}{\sum_{i=1}^{n}\frac{1}{d{\left(x_{0},y_{0},x_{i},y_{i}\right)}^{p}}}$$

where $T(x_{0},y_{0})$ denotes the meteorological element value at the target point, $T(x_{i},y_{i})$ denotes the meteorological element value at the known meteorological station, $d(x_{0},y_{0},x_{i},y_{i})$ is the spherical distance between the target point and the known point, and p is the distance weighting exponent, which is typically set to 1.5. Given that the interpolation error associated with p = 1.5 is minimal under the RMSE assessment during data processing, this value was chosen as the optimal IDW power parameter for this study. The RMSE comparison of IDW interpolation is illustrated in Figure 5.

Table 1 presents a subset of the processed data from the specific meteorological stations.

#### 3.2.2. Spatiotemporal Alignment

To align two datasets from disparate sources, the foremost step is to synchronize their timestamps. Specifically, flight trajectory data and meteorological data often exhibit discrepancies in time intervals and sampling frequencies. For instance, in the initial dataset of this study, flight trajectory data are timestamped in seconds, whereas meteorological data are recorded hourly. Addressing the challenge of data sparsity, this study opted to aggregate flight trajectory data on an hourly basis. For meteorological data, key indicators such as temperature, humidity, and wind speed were extracted and normalized. Moreover, geocoding techniques were employed to precisely match the location information of meteorological data with that of flight trajectory data, ensuring their spatial correspondence. As an example, Table 2 presents randomly selected processed data from 14 January 2025.

### 3.3. Selection of Aviation Events

In acoustics, background and industrial noises typically exhibit frequency characteristics and sources that are distinct from those of aviation noise. These noises also differ significantly from aviation noise in terms of temporal, spatial, and acoustic wave properties. If not properly addressed, background or industrial noise can significantly interfere with the prediction of aviation noise. When not distinguished, these noises blend into the dataset, causing confusion of noise sources and making it difficult for models to accurately identify and predict aviation noise. This is particularly true in noise prediction experiments, where failure to properly handle these interfering noises can lead to inaccurate results and, consequently, affect the effectiveness of noise management strategies. Ultimately, such interference not only reduces the prediction accuracy of the model but can also lead to erroneous conclusions.

**Figure 6:**
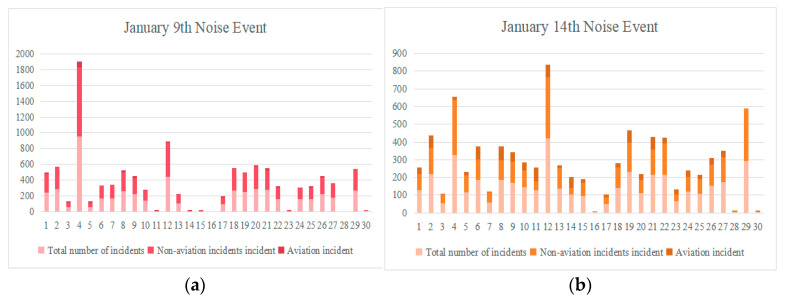
Classification of noise events at monitoring stations: (**a**) 9 January 2025; (**b**) 14 January 2025.

For noise event identification, this study integrates the timestamp information of Automatic Dependent Surveillance-Broadcast (ADS-B) data, matching the event timestamps with ADS-B data to analyze changes in data during the event, thereby further determining whether it is an aviation noise event. In the data preprocessing stage, this study screens out all aviation noise events related to flights and classifies all noise events recorded by the 30 monitoring stations over the two days of 9 January 2025, and 14 January 2025. The classification results are shown in Figure 6, with the relevant flight data used for subsequent research. Specifically, Figure 6a shows the classification of all noise events on 9 January 2025, and Figure 6b shows the classification of all noise events on 14 January 2025.

Table 3 presents the processed aviation-related events and their corresponding noise values based on the data from 9 January 2025.

### 3.4. Meteorological Influences on Noise

The generation, propagation, and reception of aircraft noise are highly complex processes, influenced by a variety of local conditions, including terrain, vegetation, and buildings, as well as meteorological factors such as humidity, wind speed, and temperature [35]. Atmospheric conditions have a decisive impact on noise levels, with spatial variations in temperature and wind speed (whether in a steady or unsteady state) causing sound wave refraction and redirecting noise waves toward areas of lower sound speed [36]. Building on this foundation, this study further integrates meteorological effects to enhance the accuracy of aircraft noise impact predictions.

1. Effect of sound speed stratification

Sound speed stratification is predominantly governed by the interplay of temperature distribution and wind speed profiles. Typically, elevated air temperatures augment the average kinetic energy of air molecules, thereby facilitating more efficient energy transfer among molecules and subsequently enhancing the velocity of sound wave propagation through the atmosphere. Concurrently, wind speed and direction exert additional influences on the effective propagation velocity of sound waves. When the wind direction coincides with the flight path of an aircraft, the sound wave propagation is effectively “boosted” by the wind, thereby extending the propagation range and expanding the noise-affected area in the downwind direction. In contrast, when the wind direction is contrary to the flight direction, sound wave propagation is hindered, resulting in a reduced propagation distance and a more localized concentration of noise.

Of paramount significance is the fact that vertical gradients in sound speed can induce refraction of sound waves. Variations in temperature or wind speed with altitude can cause sound waves to refract either upward or downward, thereby altering their trajectories as they reach the ground. Under particular meteorological conditions, such as pronounced low-level wind shear or temperature inversions, this refraction can cause noise to “linger” near the ground or propagate over unusually extended distances, thereby intensifying noise exposure in specific regions.

2. Effects of Humidity on Sound Propagation; Effects of Precipitation on Sound Propagation

Humidity, which denotes the amount of water vapor in the air, is a significant meteorological parameter influencing the propagation characteristics of sound waves. As humidity rises, the water vapor molecules in the air are lighter than the oxygen and nitrogen molecules in dry air. These lighter molecules reduce the propagation resistance of sound waves in the air, thereby increasing the speed of sound wave propagation in humid environments. Additionally, increased humidity enhances air thermal conductivity, reducing energy loss during sound wave propagation. Consequently, in high-humidity environments, sound waves attenuate more slowly and travel greater distances.

Precipitation, which refers to the total amount of liquid water in the air, including water droplets and rain, is another key meteorological factor affecting sound wave propagation. Typically, increased precipitation is associated with higher humidity. Precipitation increases the number of water droplets in the air, which absorb and scatter sound waves, accelerating their attenuation. The presence of water droplets creates more interference for sound wave propagation in the air, significantly reducing the propagation distance of sound under heavy or torrential rain conditions. Moreover, precipitation increases the inhomogeneity of the air, making the sound wave propagation path more complex. The scattering effect of water droplets can cause deviations in the direction of sound wave propagation, making sound appear muffled or weakened in certain areas.

Under real-world environmental conditions, meteorological elements typically interact with each other. For example, humid weather is often accompanied by precipitation. Therefore, assessing the impact on sound propagation requires a comprehensive consideration of the interactions among these meteorological factors.

## 4. Development of the Noise Prediction Model and Analysis of Experimental Results

### 4.1. Model Architecture

**Figure 7:**
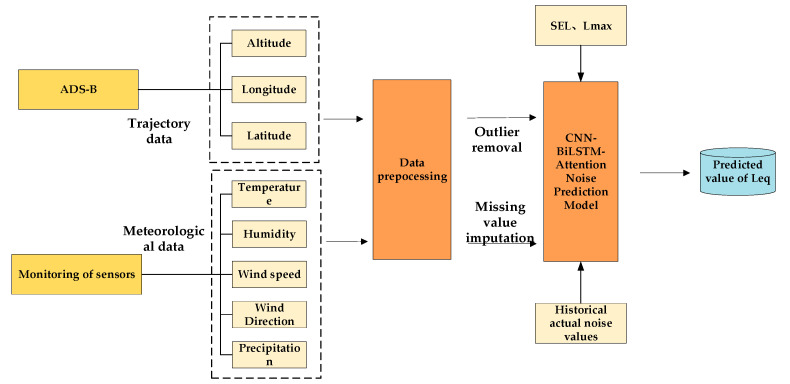
Modeling process and flowchart of the prediction model.

A Convolutional Neural Network–Bidirectional Long Short-Term Memory–Attention (CNN–BiLSTM–Attention) noise prediction model was developed using location coordinates acquired through Automatic Dependent Surveillance-Broadcast (ADS-B) and meteorological data, such as temperature, gathered by meteorological sensors. The model was constructed through a series of steps, including data preprocessing and imputation, to predict the $L_{\mathrm{Aeq}}$. The detailed process of model construction is depicted in Figure 7.

#### CNN–BiLSTM–Attention Model

Meteorological data typically appear as time series data, while trajectory data often include spatial positioning information. The CNN–BiLSTM–Attention model leverages the combined strengths of Convolutional Neural Networks (CNN), Bidirectional Long Short-Term Memory networks (BiLSTM), and attention mechanisms, enabling efficient handling of both spatial features and time series data in a concurrent manner. This integrated approach significantly enhances the model’s ability to capture both local patterns and long-term dependencies in the input data.

The original input multidimensional time series matrix is structured as depicted in Equation (5).

(5) $$X={\left[x_{1},x_{2},\ldots ,x_{T}\right]}^{T}\in R^{T\times d}$$

where T denotes the time steps; d represents the feature dimensions at each time step (such as temperature, wind speed, etc.); and $x_{t}\in R^{d}$ indicates the multi-source input vector at the *t*-th time step.

The one-dimensional convolutional layer conducts convolution operations along the temporal dimension of the input sequence, thereby capturing short-term local dynamic patterns. The specific configurations of the CNN layer are delineated in Equation (6).

(6) HCNN=ReLU(Wconv∗X+bconv)

where ∗ denotes the one-dimensional convolution operation; $W^{\mathrm{conv}}$ represents the convolution kernel parameters; $H^{\mathrm{CNN}}\in R^{T^{\prime }\times d^{\prime }}$ signifies the output feature map of the CNN; and $T^{\prime }$ is determined by the padding and stride settings.

The bidirectional LSTM effectively captures the temporal dependencies in both the forward and backward directions of the sequence, with the specific configuration of the BiLSTM layer detailed in Equation (7).

(7) $$\vec{h}=LSTM_{fwd}(H_{t}^{CNN}),\overset{\leftarrow }{h}=LSTM_{bwd}(H_{t}^{CNN})$$

The concatenation of the forward and backward hidden states is illustrated in Equation (8).

(8) $$h_{t}=[\vec{h_{t}};\overset{\leftarrow }{h_{t}}]\in R^{2h}$$

The resultant output matrix of the BiLSTM is explicitly delineated in Equation (9).

(9) $$H^{BiLSTM}={\left[h_{1},h_{2},\ldots h_{T}\right]}^{T}\in R^{T^{\prime }\times 2h}$$

The attention layer signifies the allocation of weights to individual temporal steps.

The attention scoring mechanism is delineated in Equation (10).

(10) $$e_{t}=v^{T}\tanh(W_{a}h_{t}+b_{a})$$

The configuration of the attention weights is detailed in Equation (11).

(11) $$a_{t}=\frac{\exp(e_{t})}{\sum_{i=1}^{T\prime }\exp(e_{i})}$$

The weighted summation is employed to generate a vector, as delineated in Equation (12).

(12) $$c=\sum_{t=1}^{T^{\prime }}a_{t}h_{t}$$

The output layer, which generates the noise prediction values via a fully connected layer, is detailed in Equation (13).

(13) $$\overset{\wedge }{y}=W_{0}c+b_{0}$$

where $\overset{\wedge }{y}\in R$ denotes the predicted noise metric ($L_{\mathrm{Aeq}}$).

**Figure 8:**
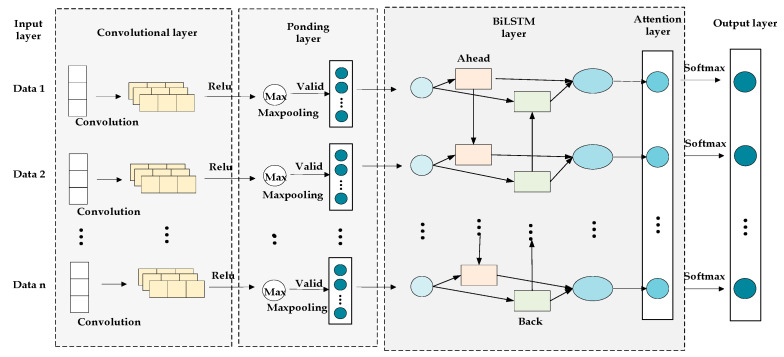
Schematic diagram of the prediction model.

The structure of the prediction model used in this study is illustrated in Figure 8, with input variables consisting of aircraft dynamic characteristics and environmental factors, and the output variable being the instantaneous A-weighted sound level at the monitoring point.

The input data encompassed the longitude, latitude, and altitude of individual flight events, as well as temperature, humidity, wind speed, and other parameters measured by associated stations at corresponding timestamps. The dataset is divided into a training set and a test set, where the first 70% of the data is used for training and the remaining 30% is used for testing. To avoid data leakage, the performance metrics of the model are evaluated only on the training set. During testing, the target noise value of the last sequence is used as a reference. The model supports a multiple-input multiple-output structure and is simplified to single-step prediction as the prediction step is set to 1 in this experiment.

Experiments were conducted to optimize the key hyperparameters of the CNN–BiLSTM–Attention model. The accuracy of noise sound pressure level predictions was assessed using mean absolute error (MAE), mean relative error (MRE), and root mean square error (RMSE).

To enhance the model’s generalization capability and mitigate overfitting risks, this study incorporates an epoch control mechanism during model training, dynamically assessing model performance across various epochs. Considering the nonlinear fitting characteristics of the CNN–BiLSTM–Attention model, we continuously monitor its validation set performance over multiple training epochs, ultimately selecting the top ten parameter configurations that yield the best performance on the validation set as model candidates. This approach effectively improves model stability and prediction accuracy.

**Figure 9:**
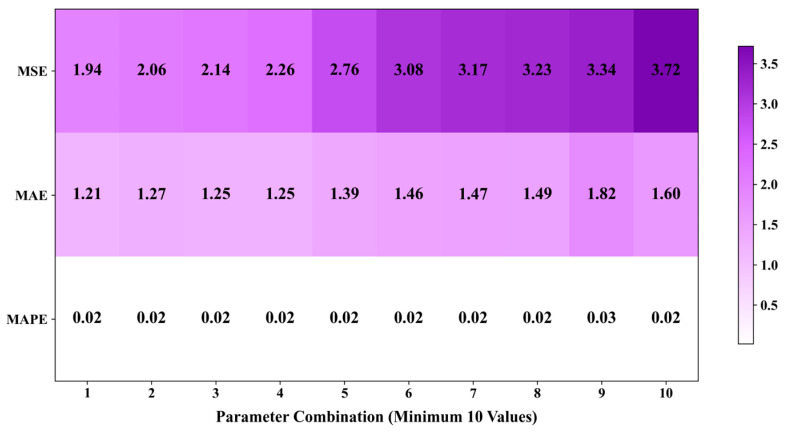
Heatmap of Experimental Evaluation Metrics.

Figure 9 presents the performance of the noise prediction model that integrates multi-source data, evaluated across three error metrics (MSE, MAE, MAPE) for various parameter combinations. The results demonstrate that the model consistently achieves low error levels across the majority of parameter combinations, thereby confirming its high prediction accuracy and stability. Specifically, the minimum Mean Absolute Error (MAE) recorded is 1.21, while the Mean Absolute Percentage Error (MAPE) remains consistently around 0.02 across all parameter combinations. These findings underscore the model’s superior ability to control relative errors. Although the Mean Squared Error (MSE) exhibits slight increases in some parameter combinations, the overall fluctuation remains minimal, indicative of the model’s robustness against outliers. In summary, the multi-source data fusion model is capable of stably generating high-quality prediction results under diverse parameter settings, thereby validating its reliability and practical applicability in noise forecasting scenarios.

### 4.2. Analysis and Discussion of Experimental Results

#### 4.2.1. Analysis of Experimental Results

During the 35 rounds of training, the performance of the predicted values of the output variable $L_{\mathrm{Aeq}}$ is shown in Figure 9. It has a high degree of agreement with the actual values. However, given the inherent characteristics of aviation noise and the interference of environmental noise, the predicted values show a certain degree of volatility.

**Figure 10:**
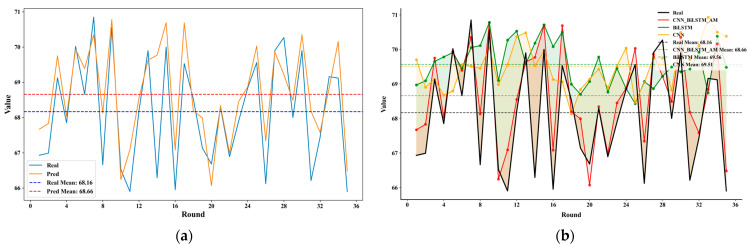
Figure of noise prediction results: (**a**) comparison of predicted values with actual observed values across multiple prediction tasks; (**b**) comparative analysis of performance metrics among CNN, BiLSTM, and the integrated CNN–BiLSTM–AM model.

Figure 10a displays the comparative analysis of the model’s predictions versus actual observed values across multiple prediction tasks. The left-hand plot highlights the temporal consistency between the actual observed values (solid blue line) and the model’s predicted values (solid orange line), demonstrating a high degree of concordance in both overall trends and fluctuation patterns. Statistical analysis reveals that the mean of the actual values is 68.16, whereas the mean of the model’s predictions is 68.66, with a minimal difference of 0.5. This finding further corroborates the model’s predictive accuracy at the overall level.

Figure 10b illustrates the ablation study conducted in this research to assess the efficacy of each module within the model’s architecture, by comparing the performance of CNN, BiLSTM, and the complete CNN–BiLSTM–AM model. The ablation results depicted on the right underscore the contributions of each structural component to the overall model performance. The findings reveal that the complete CNN_BiLSTM_AM structure yields a mean prediction value of 68.66, which is the closest to the true value, surpassing the single models utilizing only BiLSTM (69.56) or CNN (69.51). This highlights that the integration strategy, particularly after incorporating the attention mechanism, significantly enhances prediction accuracy and generalization capability. Despite minor deviations observed in some epochs, the model’s predictions consistently exhibit good dynamic alignment with the actual observed values, thereby reaffirming its reliability in handling complex multi-source data scenarios.

**Figure 11:**
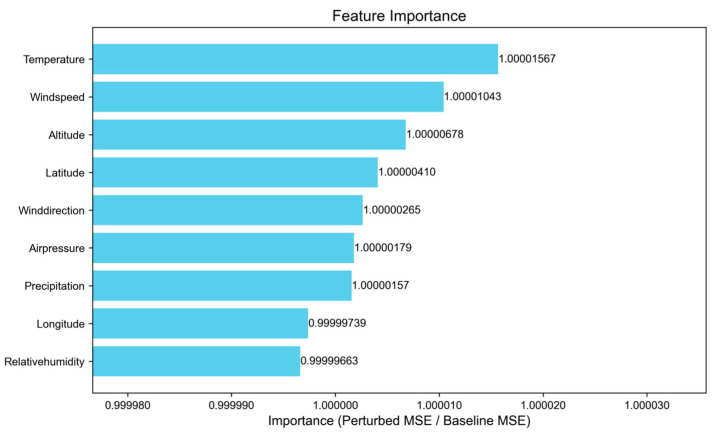
Error metrics following perturbation of key features.

To further elucidate the contribution of each meteorological feature to the model’s predictive performance, we utilized the permutation importance method. Specifically, we sequentially shuffled each input feature dimension and monitored the relative changes in model error (MSE). The results are depicted in Figure 11, where more important features are characterized by more pronounced increases in error following perturbation. The figure reveals that temperature, wind speed, and flight altitude are the most critical meteorological input factors, resulting in error amplification factors of 1.00001567, 1.00001043, and 1.00000678, respectively. Conversely, relative humidity and longitude exert a relatively minor influence on the model.

**Figure 12:**
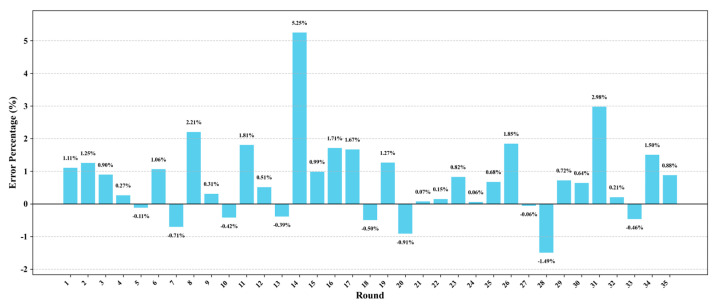
Error distribution across prediction rounds.

Figure 12 illustrates the relative prediction error percentages of the model across 35 consecutive evaluation rounds, serving to quantify the discrepancy between predicted values and actual observed values. The results reveal that the model attains high prediction accuracy in the vast majority of rounds: 91.4% (32/35) of the rounds exhibit absolute errors below 2%, with error values predominantly falling within the narrow range of −1.49% to +1.85%. This finding underscores the high stability and reliability of the prediction results overall.

Analyzing the dynamic trend of the error distribution, the model initially exhibits some fluctuation but subsequently converges progressively, thereby demonstrating robust predictive consistency. Among the total 35 rounds, 62.9% (22/35) manifest positive errors. The error distribution reveals a pattern of alternating overestimation and underestimation, with the amplitude of these fluctuations gradually diminishing to the range of −1.49% to +1.50%. This alternating pattern, in conjunction with the trend of gradual convergence, further underscores the model’s robustness and dynamic adaptability.

Moreover, the model’s Mean Absolute Error (MAE) stands at 0.89%, which underscores its capacity to sustain high precision across multiple sequence prediction tasks. The tightly clustered error distribution and minimal fluctuations collectively corroborate the proposed model’s accuracy, stability, and robust generalization adaptability in extended time series forecasting endeavors.

#### 4.2.2. Applicability and Limitations of the Model Under Sparse Sampling Conditions

The collection of data for aviation noise prediction often requires sophisticated instruments and entails significant costs, particularly when acquiring data in real-world settings. These requirements are frequently constrained by limitations in resources and time, which greatly increase the difficulty of data collection. Additionally, the aviation sector involves a substantial amount of sensitive information, much of which is subject to strict privacy protections and regulations that preclude public sharing. This further restricts the availability and application of data. As a result, in specific contexts, the accessible data resources are relatively limited, contributing to data scarcity.

Under ultra-sparse data conditions, deep learning models often suffer from overfitting and inadequate learning of underlying patterns. To mitigate these challenges, this study employs interpolation techniques to fill missing data, designs rational feature engineering strategies, and applies L2 regularization and dropout to reduce model complexity. The CNN–BiLSTM–Attention architecture demonstrates strong robustness and generalization in such limited-data scenarios. CNN effectively extracts local spatial patterns, BiLSTM captures bidirectional temporal dependencies, and the attention mechanism emphasizes critical time steps. Experimental results confirm that even with a small sample size, the integrated model maintains relatively high prediction accuracy for aviation noise, highlighting its effectiveness under data scarcity.

## 5. Conclusions

As the global population continues to grow and urbanization accelerates, aviation noise pollution has emerged as a significant environmental challenge, impeding the sustainable development of the aviation industry. Traditional noise prediction techniques are constrained by incomplete datasets, insufficient spatiotemporal consistency, and poor adaptability to complex meteorological conditions, making it difficult to meet the stringent requirements of precise noise management. To address these limitations, this study introduces a sparse sample noise prediction framework based on Convolutional Neural Networks (CNN) and Bidirectional Long Short-Term Memory networks (BiLSTM) with Attention. By integrating multi-source data, including meteorological parameters and aircraft trajectory data, the framework achieves high-precision prediction of aviation noise. During the research process, the Haversine formula and inverse distance weighting (IDW) interpolation method were employed to effectively supplement missing data, and spatiotemporal alignment techniques were used to ensure data consistency. Moreover, the CNN–BiLSTM–Attention model combines the spatial feature extraction capabilities of CNNs, the bidirectional temporal sequence processing expertise of BiLSTMs, and the context-enhancing properties of Attention, effectively capturing the spatiotemporal characteristics of noise. The experimental results indicate that the model’s mean prediction value is 68.66, deviating by a mere 0.5 from the actual value of 68.16, and achieving a Mean Absolute Error (MAE) of 0.89%. Notably, 91.4% of the prediction rounds feature errors below 2%. Additionally, the maximum value of the Mean Absolute Percentage Error (MAPE) is 0.03. These results demonstrate the model’s strong performance. This study not only offers a scientific foundation for effective aviation noise management but also presents an innovative approach to addressing the challenge of noise prediction in data-scarce conditions.

The findings of this study provide a scientific basis for aviation noise management and offer innovative solutions for addressing noise prediction problems under data-scarce conditions. Through multi-source data fusion and spatiotemporal feature modeling, the framework constructed in this study can comprehensively analyze the spatiotemporal distribution characteristics of noise, providing robust data support for the formulation of airport noise control policies. Additionally, the framework proposed in this study has broad applicability, not only to specific airports but also extendable to other environmental noise monitoring fields, such as urban traffic noise and industrial noise. By integrating multiple data sources, a comprehensive analysis of noise spatiotemporal distribution and correlation characteristics can be conducted, offering a new technological pathway for environmental noise monitoring.

Despite significant academic progress in the field of noise prediction, several limitations remain. For instance, the layout of monitoring points is constrained by terrain conditions and equipment costs, which may affect the global accuracy of the interpolation model. Future research can further optimize the layout of monitoring points by combining Geographic Information Systems (GIS) and terrain analysis to rationally plan their locations, ensuring the representativeness and comprehensiveness of the data. Moreover, the nonlinear relationship between meteorological conditions and noise propagation can be further modeled and optimized. Future studies could consider incorporating machine learning algorithms (e.g., spatial random forests) to enhance the generalization ability of interpolation models, thereby improving their accuracy and robustness. Exploring three-dimensional noise distribution modeling to support vertical space analysis will also help more comprehensively reflect the propagation patterns of noise at different altitudes.

In summary, this study has achieved technological innovation and demonstrated its effectiveness in practical applications. By integrating multi-source data and constructing spatiotemporal feature models, this study offers an innovative methodology for the field of aviation noise monitoring. The results are not only applicable to specific airport environments but also scalable and can be extended to other airports and environmental monitoring fields. Further research and application are expected to contribute more theoretical and practical support to the advancement of aviation noise management and environmental noise monitoring technologies.

## Figures and Tables

**Table 1:** Table of processed meteorological data.

Date Time (mm/dd/hh:mm)	Wind Speed (km/h)	Wind Direction (°)	Temperature (°C)	Air Pressure (pa)	Relative Humidity (%)	Precipitation (mm)
2025/1/9 3:00	0.26	7.001	7.479	991.203	88.368	10.6
2025/1/9 4:00	0.189	7.001	7.367	990.895	88.431	10.597
2025/1/9 5:00	0.695	7	7.286	991.24	89.145	10.6
2025/1/9 6:00	0.665	7.001	7.286	991.178	89.38	10.6
2025/1/9 7:00	0.208	7.001	7.376	991.589	89.851	10.596
2025/1/9 8:00	0.245	7.001	7.515	992.787	88.23	10.594
2025/1/9 9:00	0.282	7.001	7.865	993.506	85.621	10.597
2025/1/9 10:00	0.253	7.001	8.128	994.718	83.604	10.597
2025/1/9 11:00	0.223	7.001	8.122	994.723	83.263	10.6
2025/1/9 12:00	0.194	7	7.548	994.617	88.555	10.6
2025/1/9 13:00	0.301	7.001	7.64	992.165	87.743	10.585
2025/1/9 14:00	0.13	7.001	7.797	992.711	87.342	10.591
2025/1/9 15:00	0.593	7.001	8.146	992.538	85.446	10.591
2025/1/14 9:00	0.05	7.001	8.914	991.742	86.9	10.591
2025/1/14 10:00	0.024	7	9.109	992.062	86.328	10.591
2025/1/14 11:00	0.082	7.001	9.033	992.167	86.554	10.593
2025/1/14 12:00	0.141	7.001	8.956	992.272	86.781	10.595
2025/1/14 13:00	0.199	7.002	8.88	992.377	87.007	10.597
2025/1/14 14:00	0.91	7	9.076	991.809	84.76	10.597
2025/1/14 15:00	0.755	7.002	9.292	992.401	84.329	10.597
2025/1/14 16:00	0.547	7.001	9.33	992.918	84.773	10.597
2025/1/14 17:00	0.339	7.001	9.368	993.435	85.218	10.597
2025/1/14 18:00	0.131	7	9.406	993.952	85.662	10.597
2025/1/14 19:00	0.131	7	9.406	993.952	85.662	10.597
2025/1/14 20:00	0.131	7	9.406	993.952	85.662	10.597
2025/1/14 21:00	0.131	7	9.406	993.952	85.662	10.597

**Table 2:** Table of spatiotemporally aligned data.

Longitude (°)	Latitude (°)	Altitude (m)	Date Time (mm/dd/hh:mm)	Flight Number	Wind Speed (km/h)	Temperature (°C)	Air Pressure (pa)	Relative Humidity (%)
106.6672683	29.78231549	42.25	2025/1/14 11:37	HO1692	0.018	8.336	987.023	90.442
106.6751003	29.75416839	22.25	2025/1/14 11:39	CZ5754	0.018	8.336	987.023	90.442
106.6747034	29.75338519	23.75	2025/1/14 11:44	CZ6314	0.018	8.336	987.023	90.442
106.6537499	29.74121869	24.25	2025/1/14 11:57	MU2926	0.018	8.336	987.023	90.442
106.6539055	29.74305332	21.25	2025/1/14 12:17	DZ6285	0.033	8.469	986.91	89.667
106.6508585	29.73455071	15.25	2025/1/14 13:03	G54121	0.048	8.602	986.798	88.893
106.6565502	29.74861622	25	2025/1/14 13:09	3U8831	0.048	8.602	986.798	88.893
106.65088	29.73406255	14	2025/1/14 13:12	MU2330	0.048	8.602	986.798	88.893

**Table 3:** Table of processed aviation-related events.

Date_Time (mm/dd/hh:mm)	Flight_Number	Monitor_Station	Leq (dBA)	SEL (dBA)	Lmax (dBA)
2025/1/9 0:09	G54262	8	67.2	80.42	72
2025/1/9 1:38	3U8062	9	68.42	81.64	72.8
2025/1/9 1:50	PN6230	9	67.53	80.08	72.3
2025/1/9 1:53	PN6438	9	67.29	79.84	71.8
2025/1/9 2:20	CA4384	8	69.28	80.07	75.9
2025/1/9 3:59	OQ2394	8	67.81	80.12	73.7
2025/1/9 7:02	HU7342	27	66.16	78.46	72.4
2025/1/9 7:27	G52875	4	87.67	105.52	87.7
2025/1/9 7:34	OQ2341	1	69.43	84.34	73.5
2025/1/9 8:30	G54180	8	69.17	82.18	74.9
2025/1/9 8:30	G54180	9	67.35	81.33	72.6
2025/1/9 8:31	G54009	1	69.09	82.71	74.8
2025/1/9 8:33	9C8613	9	68.03	80.59	73.8
2025/1/9 9:09	MU2903	8	67.69	80.48	73.9
2025/1/9 9:26	CZ3620	27	68.91	82.33	75.3
2025/1/9 10:24	KN5269	9	68.13	80.17	75.2
2025/1/9 13:32	HU7341	8	69.78	81.24	77
2025/1/9 14:21	OQ2327	18	68.15	82.92	72.4
2025/1/9 14:37	HO2004	4	94.27	99.04	95.5
2025/1/9 14:45	LT4319	9	67.73	81.54	72.7

## Data Availability

The original contributions presented in this study are included in the article. Further inquiries can be directed to the corresponding author.
